# Knowledge and awareness on pneumoconiosis among dental technicians and senior dental technology students in Saudi Arabia

**DOI:** 10.6026/97320630018968

**Published:** 2022-10-31

**Authors:** Abbasi Begum Meer Rownaq Ali, Haifa Sultan Dathan Alharbi, Bushra Faraj Sahmi Al-Subaie, HM Khuthija Khanam, Hina Kausher

**Affiliations:** 1 Department of Prosthodontics, Dental Lab Technology, Riyadh Elm University, Riyadh - Kingdom of Saudi Arabia; 2 Dental Lab Technology, College of Applied Medical Sciences, Riyadh Elm University, Riyadh - Kingdom of Saudi Arabia; 3 Dental Lab Technology, College of Applied Medical Sciences, Riyadh Elm University, Riyadh - Kingdom of Saudi Arabia; 4 Department of Restorative and Prosthetic Dental Sciences, Dar Al Uloom University, Riyadh, Kingdom of Saudi Arabia; 5 Department of Prosthodontics, Dental Lab Technology, College of Applied Medical Sciences, Riyadh Elm University, Riyadh- Kingdom of Saudi Arabia

**Keywords:** Pneumoconiosis, dental technicians, respiratory disorders

## Abstract

Dental technicians' pneumoconiosis is a condition that causes interstitial inflammation and fibrosis as a result of complex drug exposure. Therefore, it is of interest to assess and evaluate the knowledge and awareness about pneumoconiosis and other respiratory disorders among dental technicians and dental technology students in Saudi Arabia. A cross sectional survey was conducted on convenient sample of 300 senior dental technician students and dental technicians from Saudi Arabia and informed consent was obtained. The participants were approached through online questionnaire using Google forms. Questions were related to the socio-demographic data, knowledge and awareness of the students and the practicing dental technicians and their willingness to learn about pneumoconiosis. Final year undergraduate, interns and Dental technicians were included, undergraduate students without practical knowledge and experience and participants with history of respiratory disease were excluded from the study. Questions were assessed by face validity. The data was analyzed using Statistical Package for Social Sciences software. Descriptive statistics was calculated. 34.8% of the study group were not aware of the term and don't know about the etiology of pneumoconiosis. Pearson correlation was significant in terms of awareness difference between males and females and females had less awareness compared to males.

## Background:

Dental laboratory technicians work with a variety of harmful compounds, such as silica used as an abrasive in cabinet-style sandblasters and as a component of porcelain, as well as non-precious metal alloys used to make crowns, bridges, and dentures [1]. Pneumoconiosis is a category of severe occupational illnesses brought on by mineral dust inhalation and the lung tissue reaction that results in permanent lung damage. [2, 3,4]. Due to exposure to complicated chemicals, dental technicians are more likely to develop interstitial inflammation and fibrosis [5, 6]. It can result in severe health issues and persistent respiratory failure, which raises the chance of complications from tuberculosis and lung cancer [4, 7]. Pneumoconiosis instances have so far been reported in a number of cases. [3, 6, 8,9]. The main factor in preventing this condition, which is brought on by exposure to mineral dust and fibers, is adherence to workplace exposure management and health management, which are governed by the pertinent regulatory framework. [10] Workplace health management entails monitoring a worker's continuing health in order to detect abnormalities as soon as possible and take the required action to stop illness onset and progression. [11]. Pneumoconiosis is a serious health risk for dental technicians [6] and is typically brought on by inhaling a mixture of dusts, including crystalline silica and hard metals like chromium-cobalt-molybdenum alloys [3, 12]. Therefore, it is of interest to assess the knowledge and awareness of pneumoconiosis among dental technology students and working dental technicians in Saudi Arabia.

## Methodology:

## Sample Selection:

 A sample of 300 senior dental technician students and practicing dental technicians (including both males and females) from various dental universities and dental laboratories in Saudi Arabia were selected.

## Informed consent:

After obtaining approval from the Institutional ethical committee Review Board, Riyadh Elm University, IRB approval number "FUGRP/2021/272/696/670" in compliance with codes of research ethics, an informed consent was obtained from research participants.

## Measurement:

The participants were approached through a structured online questionnaire using Google forms. The questionnaire was divided into three main parts, the first part was about the socio-demographic behavioural items including age, gender, students' current level, city/region, University, second part deals with more detailed questions related to the knowledge of the students and the practicing dental technicians with regards to pneumoconiosis and Questions related to their awareness and their willingness to learn were included in the 3rd section.

## Inclusion and exclusion criteria:

Students with higher levels of practical Knowledge: final year undergraduate and interns and dental technicians were included. Undergraduate students without practical knowledge and experience and patients with previous history of respiratory disease were excluded from the study.

## Validation of questionnaire:

Questions in the Questionnaire were assessed for face validity by an experienced Dental technician and Dentist.

## Statistical analysis:

Software from the Statistical Package for Social Sciences (SPSS) was used to code, tabulate, and analyze the data. We then calculated descriptive statistics.

## Results:

 Among the study participants majority (47%) were around 21-25 years of age and 80% were males and around 48.7% of the participants were technologists. (Table 1-see PDF) Only 39.5% of the study group were aware of the term pneumoconiosis. Nearly half of the study group didn't know the etiology of pneumoconiosis and 27.1% have never heard about Pneumoconiosis. The source of information about pneumoconiosis for the majority of the study group is from social media followed by research articles and least from the books (only 10%). (Table 2-see PDF) When comparing various educational levels, it was found that books as a source of information has steadily declined and this signifies the substantial need for conducting continued education programs for creating awareness among the dental technicians to keep oneself aware of various ill effects of respiratory diseases associated with their occupation as well as substantial need for focusing on theoretical and practical courses covering overall aspect of respiratory diseases as a part of undergraduate curriculum of the dental technology program. (Table 3-see PDF) Less than 50% of the study group knew that dental technicians are more prone to develop pneumoconiosis. Also, more than 58.8% of the study group didn't know that pneumoconiosis will eventually induce irreversible lung damage. Pneumoconiosis in dental technicians can be caused by exposure to dust with high silica concentrations and cobalt-chromium molybdenum alloys, however more than half of the study population was unaware of this and was unsure of how to prevent it. Around 30.2% have suffered from dyspnea during the lab work and about 57.8% have responded that mixing and processing of acrylic is the cause for difficulty in breathing followed by mechanical spatulation of gypsum products (20%), acrylic trimming (10.4%), trimming and polishing of metal crowns (7%) and cast partial dentures trimming and polishing around 4.8%. (Table 4-see PDF) The Pearson co-relation with respect to the difference in the knowledge between males and females with respect to question on technicians being more prone to pneumoconiosis and also their response to the etiology of dust exposure is significant. Also, the study shows the level of awareness about pneumoconiosis is greater among males when compared to females with statistically significant (≤0.05) Pearson correlation value. (Table 5-see PDF) Also, the response to the question on pneumoconiosis inducing irreversible lung damage the Pearson correlation with respect to the knowledge between various educational levels and gender was statistically significant (Table 6-see PDF).

## Discussion:

Pneumoconiosis and other respiratory illnesses is the subject of a dearth of studies among Saudi Arabia's dental technicians. Only 39.5% of the study sample knew what the term "pneumoconiosis" meant, according to the study's restrictions. We discovered that 30.2% of dental technicians experience dyspnea when working in the lab as a result of ignorance and lack of awareness. Additionally, studies indicate that pneumoconiosis patients have higher rates of lung cancer and tuberculosis than the general population [6, 13,14], which is a worrying indication that early diagnosis, knowledge of risk factors, and preventive measures are essential to lowering the disease's mortality and morbidity rates. It is frequently observed that dental professionals prefer not to wear personal protective equipment since it may limit their ability to perform precise work [7]. While 83.7% of respondents to the current study believe that using a combination of face mask, eyeglasses, face shield, and vacuum installed devices can protect them from dust during lab work, 6.7% believe that using a face mask alone, 2.7% that wearing a face shield and eyeglasses alone, 4.3% that using a vacuum installed device, and 2.7% that using no protective measures can protect them from dust. The majority of self-employed dental technicians operate without ventilation for more than eight hours a day, which puts them at a higher risk for occupational lung diseases, according to a study by Choudat et al. A common issue in dental laboratories is inadequate exhaust ventilation. When improperly ventilated, several dust constituents, especially those produced during grinding, have been found to drastically exceed requirements [12]. It is concerning to note that more than half of the participants in the current study were unaware that exposure to dust containing high silica concentrations and cobalt-chromium molybdenum alloys increases the risk of dental technicians developing pneumoconiosis, which emphasises the importance of installing an appropriate dust control system and ventilation. Employee health is continuously monitored as part of workplace health management in order to identify irregularities early and take the necessary action to stop illness occurrence as well as to provide the treatment required to stop disease development [11]. According to studies, pulmonary function tests and radiological tests, such as chest X-rays or high-resolution computerised tomography (HRCT), should be done in conjunction to assess occupational exposure in dental technicians [15,16] Numerous studies have shown that HRCTs are more sensitive than chest radiography at detecting small opacities, interstitial lung fibrosis, and emphysema in patients of low grade pneumoconiosis [15,17, 18]. Therefore, it is important to inform dental laboratory professionals of the value of undergoing HRCT in order to identify radiological alterations associated with occupational lung disease at their earliest stages. A case study conducted by Tiraboschi et al. in 2015 of a dental technician who developed a respiratory symptom shortly after beginning work and diagnosed with pneumoconiosis was due to abnormal occupational exposure to metals. Mineralized dust, such as silica, aluminium oxide, asbestos fibres, and various metals like cobalt chromium, beryllium, and nickel alloys, produced during the fabrication of dental prosthetics can cause lung fibrosis and serious lung conditions like pneumoconiosis, hypersensitivity pneumonitis, lung granulomatosis, asthma, and lung cancer [5]. Progressive extensive fibrosis or complex pneumoconiosis can both lead to impaired pulmonary function. [16,17, 18,19,20] In addition to respiratory risks, Dental technicians are susceptible to dermatitis. Due to these negative impacts, it is vitally important to inform dental lab technologists and students about the same. At the technician's workstations, dust concentration in dental laboratories should be measured and should not be allowed to exceed the danger limits. The health issues associated with exposure to dust should be made aware to all dental lab workers and workplace management, and working conditions must be managed. Dental technicians are willing to learn about pneumoconiosis in 92.9% of cases. The majority of dental technicians believe that wearing a face shield, mask, and utilising a dust extractor are the best ways to prevent accidents because they deal with dangerous materials on a daily basis.

## Conclusion:

Data shows that 34.8% of the study participants were aware of the term and don't know about the etiology of pneumoconiosis. Pearson correlation was significant in terms of awareness difference between males and females and females had less awareness compared to males. None of the senior technologist has learnt about it from books and majority of them have heard about pneumoconiosis from media. It was concluded that there is not enough knowledge and awareness regarding pneumoconiosis and it is recommended that there is a substantial need for introducing dedicated theoretical and practical courses covering all aspects of respiratory diseases as a part of undergraduate curriculum of the dental technology program and conducting regular continued education programs for dental technicians emphasizing the significance of using preventive measures to avoid serious health complications. Routine health screening should be carried out to detect pneumoconiosis, a serious occupational condition that affects dental technicians, early.

## Recommendations:

1) Prevention of this disease-compliance with workplace exposure management and health management regulated within relevant legal framework

2) There is a substantial need for focusing on theoretical and practical courses covering overall aspect of respiratory diseases as a part of undergraduate curriculum of the dental technology program

3) Regular health screenings are recommended for early detection of pneumoconiosis, one of the cardinal occupational condition among dental technicians.

4) In dental laboratories, adequate local ventilation system which has a critical role in reducing the air contaminants should be considered to avoid dental technicians from being exposed to airborne pollutants.
